# Anterior Dural Tear in Thoracic and Lumbar Spinal Fractures: Single-Center Experience with Coating Technique and Literature Review of the Available Strategies

**DOI:** 10.3390/life11090875

**Published:** 2021-08-25

**Authors:** Giorgio Lofrese, Jacopo Visani, Francesco Cultrera, Pasquale De Bonis, Luigino Tosatto, Alba Scerrati

**Affiliations:** 1 Ospedale “M. Bufalini” di Cesena, University of Ferrara, 44100 Ferrara, Italy; francesco.cultrera@auslromagna.it (F.C.); luigino.tosatto@auslromagna.it (L.T.); 2 Department of Morphology, Surgery, and Experimental Medicine, Arcispedale Sant’Anna, University of Ferrara, 44100 Ferrara, Italy; jacopo.visani@student.unife.it (J.V.); dbnpql@unife.it (P.D.B.); scrlba@unife.it (A.S.)

**Keywords:** anterior, ventral, dural tear, CSF leakage, repair, watertight, patch, graft, spinal fracture

## Abstract

Differently from the posterior, the anterior dural tears associated with spinal fractures are rarely reported and debated. We document our experience with a coating technique for repairing ventral dural lacerations, providing an associated literature review on the available strategies to seal off such dural defects. A PubMed search on watertight repair techniques of anterior dural lacerations focused on their association with spinal fractures was performed. Studies on animal or cadaveric models, on cervical spine, or based on seal/gelfoam or “not suturing” strategies were excluded. 10 studies were finally selected and our experience of three patients with thoracic/lumbar spinal fractures with associated ventral dural tear was integrated into the analysis of the surgical techniques. Among the described repair techniques for ventral dural lacerations a preference for primary suturing, mostly trans-dural, was noted (*n* = 6/10 papers). Other documented strategies were the plugging of the dural opening with a fat graft sutured to its margins, or stitched to the dura adjacent to the defect, and the closure of the dural tear with two patches, both trans-dural and epidural. Our coating techniques of the whole dural sac with the heterologous patch were revealed as safe and effective alternatives strategies, even when patch flaps wrapping nerve roots have to be cut and a fat graft has to be stitched in the patch respectively for sealing off antero-lateral and wide anterior dural tears. Compared to all the documented strategies for obtaining a watertight closure of an anterior dural laceration, the coating techniques revealed advantages of preserving neural structures, being adaptable to anterior and antero-lateral dural tears of any size.

## 1. Introduction

Dural lacerations are relatively common findings in thoracic and lumbar spinal fractures. Usually posterior and sustained by fractured laminae, dural tears are typically associated with burst fractures and easily identified through a standard posterior approach. Nevertheless, few reports discuss anterior dural lacerations, registered in up to 10% of patients with thoracic and lumbar burst fractures, and resulting from vertebral body fragments encroaching the spinal canal [1]. Often missed, due to their location being difficult to detect through the conventional posterior approach, the repair of such ventral dural tears is a challenge. Herein we present our experience with a coating technique and an overview of the literature on different surgical strategies for sealing off anterior dural lacerations in a watertight fashion.

## 2. Materials and Methods

### Literature Review

A PubMed search (filters: English language, human subjects) up to March 2021 was performed by using the terms “dural tear”, “dural laceration”, “dural injury”, and “dural leakage”, in association with the following major MeSH headings with the Boolean operator “AND” and “OR” in all the possible combinations of research: “anterior” (*n* = 101 papers), “ventral” (*n* = 27 papers), “fracture” (*n* = 128 papers), “spine fracture” (*n* = 3 papers), “spinal fracture” (*n* = 4 papers), “vertebral fracture” (*n* = 5 papers), “spine trauma” (*n* = 2 papers), “spinal trauma” (*n* = 9 papers), “spine injury” (*n* = 3 papers), “spinal injury” (*n* = 10 papers), “repair” (*n* = 141 papers), “closure” (*n* = 44 papers), “management” (*n* = 142 papers), and “technique” (*n* = 160 papers). After duplicates removal, papers were further filtered excluding cadaveric studies, manuscripts on animal models, works on dural tears of the cervical spine, dural closures exclusively with sealant and gel-foam, repairs without suturing the dural patch (autologous or heterologous) and all those manuscripts not specifying the duraplasty procedure.

Papers describing repair techniques for anterior dural lacerations as consequences of degenerative spine disease or surgical manipulation were not excluded, both because the mentioned strategies could be adopted even in post-traumatic ventral dural tears, and since one of the purposes of this paper is collection of all the documented techniques aimed at restoring a closed dural compartment as watertight as possible. Dural tears along the midline of the anterior dural surface and on the antero-lateral dura close to the nerve root were respectively defined as “anterior” and “antero-lateral”.

## 3. Coating Techniques

### 3.1. Dural Coating

**Figure 1:**
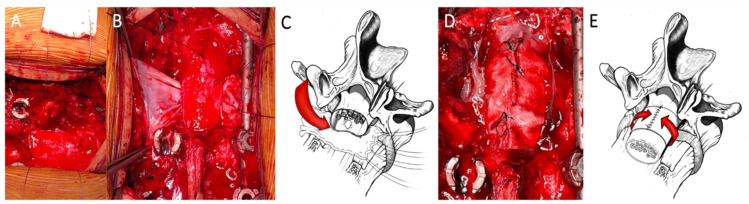
Coating technique. By pulling the threads (**A**), the patch slides under the dural sac reaching a position covering all the anterior dural surface from side to side (**B**,**C**). After having properly centered the patch on the tear and having folded its flaps onto the dorsal dural surface, a running suture for closing the entire wrap is performed (**D**,**E**).

After the exposure of the dural tube, a blunt dissection of its ventral surface from the posterior wall of the vertebral body is performed, a sterile centimeter is inserted anteriorly surrounding the dural sac, and measures are taken for shaping a patch (Tutopatch^®^), adequately cut to entirely envelop the dural tube. The shaped patch is pinched at its angles by suture threads and these are made to slide flush to the posterior wall of the vertebral body. By pulling the threads, the patch slides under the dural sac reaching a position covering all its anterior surface from side to side. After having properly centered the patch on the tear and having folded its flaps onto the dorsal dural surface, a running suture to close the entire wrap is performed, being careful not to constrict the dural tube underneath by tightening the margins of the patch too much with the suture (Figure 1).

### 3.2. Dural Coating Straddling Nerve Roots

**Figure 2:**
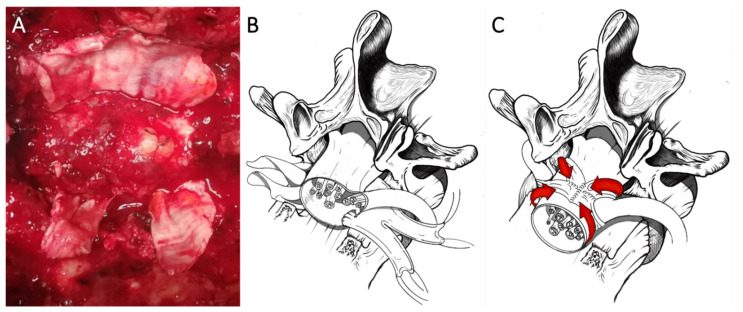
Coating technique straddling nerve roots. Intraoperative image showing the dural sac, previously covered with a stitched fat graft, and the dural patch ready to be folded onto the dorsal dural surface (**A**). By pulling the threads, the patch slides under the dural sac, reaching a position covering the dural tear, and two cuts are made to form a slot long enough to accommodate both the nerve roots without retracting them (**B**). After having folded the flaps of the patch onto the dorsal dural surface, a running suture to close the entire wrap is performed, being careful not to constrict the nerve roots (**C**).

Exploiting the same procedure aimed at dissection of the anterior surface of the dural sac from the posterior wall of the vertebral body and to measure the circumference of the dural tube for shaping a tailored patch, this latter is pinched at its angles by suture threads and is made to slide flush to the posterior wall of the vertebral body by pulling the threads. The patch slides under the dural sac reaching a position covering all its anterior surface from side to side. The Tutopatch^®^ is adequately positioned on the antero-lateral dural tear close to the nerve root axilla and two cuts are made to form a slot long enough to accommodate the nerve roots of both sides without retracting them. After having folded the flaps of the patch onto the dorsal dural surface, a running suture to close the entire wrap is performed, being careful not to constrict the nerve roots tightening too much the suture of the loop of the dural patch surrounding them as a scarf (Figure 2*).*

### 3.3. Dural Coating with Sutured Fat Graft

**Figure 3:**
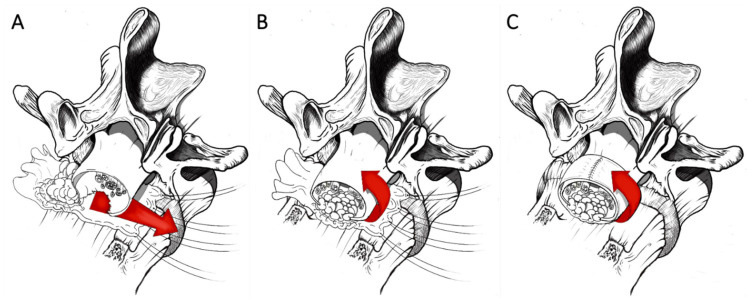
Coating technique with sutured fat graft. An autologous fat graft (2.5 cm × 2.5 cm) harvested from the subcutaneous tissue is stitched at the geometric center of the patch and this latter pinched at its angles by suture threads (**A**). By pulling the threads, the patch slides under the dural sac reaching a position covering all the anterior dural surface from side to side and perfectly plugging the dural gap with its stitched fat graft (**B**). After having folded the flaps of the patch onto the dorsal dural surface, a running suture to close the entire wrap is performed (**C**).

After the usual blunt dissection of the ventral surface of the dural sac from the posterior wall of the vertebral body, a sterile centimeter is inserted anteriorly surrounding the dural tube, and measures are taken to shape a patch (Tutopatch^®^). An autologous fat graft harvested from the subcutaneous tissue and with an adequate volume to plug the dural defect is stitched at the geometric center of the patch and this latter is pinched at its angles by suture threads. By sliding these flush to the posterior wall of the vertebral body and by pulling the threads, the patch slides under the dural sac reaching a position covering all the anterior dural surface from side to side and perfectly plugging the dural gap with its stitched fat graft. After having folded the flaps of the patch onto the dorsal dural surface, a running suture for closing the entire wrap is performed with the same precautions as for the standard dural coating (Figure 3).

## 4. Results

### 4.1. Case Report 1: Coating

**Figure 4:**
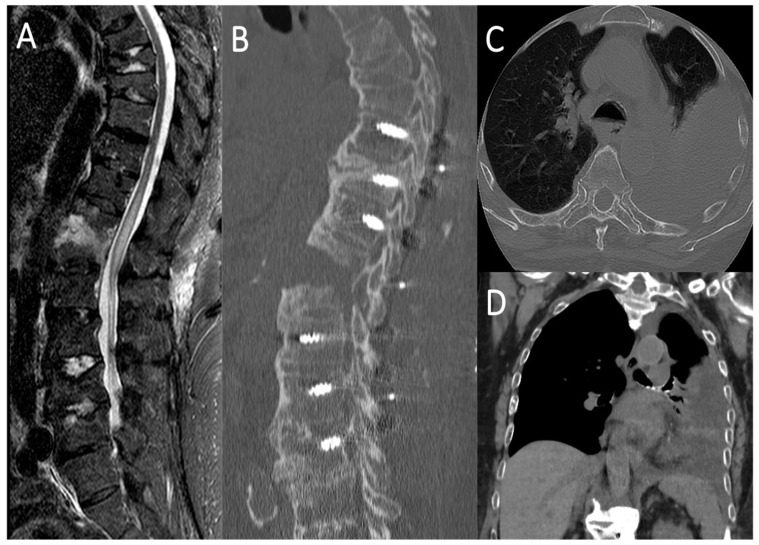
Spine MRI showing the T12 type B3 fracture with myelopathy of the conus medullaris (**A**); Post-operative CT-scan documenting the T9-L3 screw-rod fixation (**B**); Axial (**C**) and coronal (**D**) view of thoraco-abdominal CT-scan showing the massive left-sided pleural effusion.

A 78 year-old female was involved in a domestic accident, with a fall from standing height. Acute neurological examination revealed severe motor impairment of the lower extremities and neurogenic bladder, i.e., an American Spinal Cord Injury Association impairment scale (ASIA): A. Urgent spine CT-scan documented a T12 type B3 fracture (AOSpine thoracolumbar classification system, Table 1) [2]. MRI revealed a post-traumatic myelopathy of the conus. Patient underwent urgent surgery through a T9-L3 transpedicular screw-rod fixation and the early post-operative course was uneventful. Then, 18 days after surgery, due to a sudden dyspnea and desaturation, the patient underwent an urgent chest X-ray and a thoraco-abdominal CT-scan, which detected a massive left-sided pleural effusion (Figure 4). A left thoracentesis was performed with a partial relief of respiratory symptoms. The analysis of the pleural fluid drained confirmed the suspicion of cerebrospinal fluid (CSF) leakage in the pleural space through the beta-2-transferrin test. A spinal catheter for CSF drainage was positioned for 5 days, but since the dyspnea did not improve, with a worsening of the pleural effusion at chest X-ray control, the spinal catheter was removed and one week after surgery the patient was re-operated upon. A T12 laminectomy was performed, and the T12 level of CSF leakage was intraoperatively verified noting a CSF outflow from the ventral surface of the dural sac. Due to its anterior location the dural tear was impossible to identify in its whole length without twisting and retracting the dural tube. This latter was ventrally dissected from the posterior wall of the vertebral body. A dural patch (Tutopatch^®^), adequately cut, was first positioned to enfold the dural sac, then closed with a running suture dorsally. The dural coating was deemed watertight to Valsalva maneuver before closure of the fascia and skin. In 5 days, the pleural effusion sustained by the CSF leakage gradually improved and on the sixth post-operative day the patient was discharged. Patient was available up to 15 months at follow up, reporting the resolution of respiratory symptoms, the complete healing of the surgical wound, but the persistence of the pre-operative neurological impairment.

### 4.2. Case Report 2: Coating Straddling Nerve Roots

**Figure 5:**
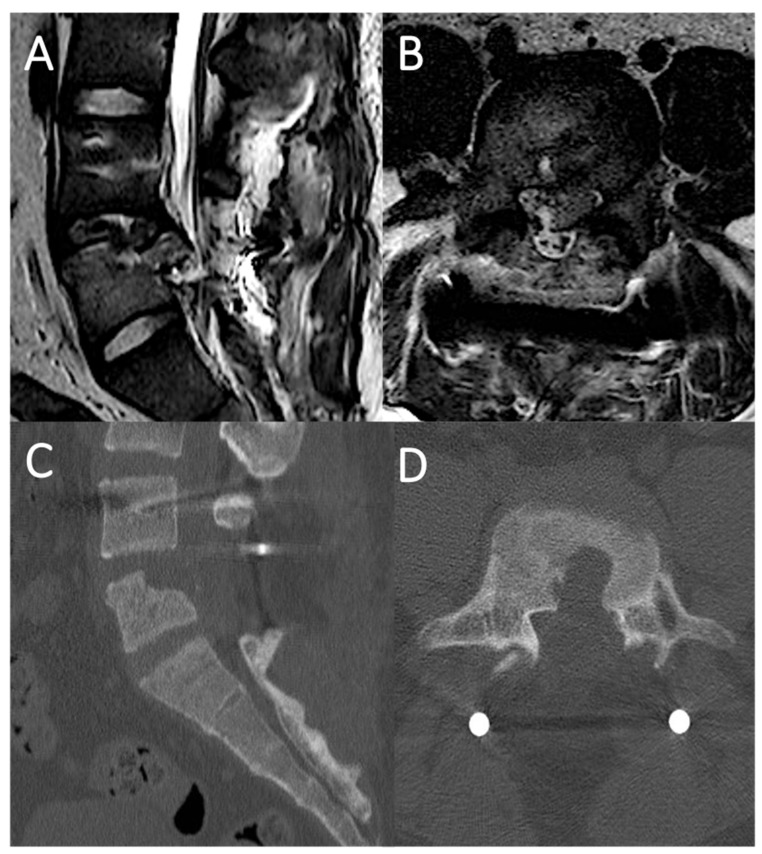
Lumbosacral spine MRI showing the L5 type A3 fracture with a single vertebral body fragment piercing the dural sac in sagittal (**A**) and axial view (**B**); Post-operative CT-scan after the revision surgery documenting the vertebral body fragment removal in sagittal (**C**) and axial view (**D**).

An 18 year-old male was involved in a car accident. No impairments were documented at the neurological examination (ASIA impairment scale: E). Urgent spine CT-scan showed a L5 type A3 fracture with a single vertebral body fragment encroaching the spinal canal by over 50%. Patient underwent urgent surgery through a L4-sacrum screw-rod fixation and L5 laminectomy to provide stability to the fractured vertebra and decompress the nerve roots. The early post-operative course was uneventful, but on the fourth post-operative day a CSF leakage from the surgical wound occurred. A revision surgery was performed without intraoperative identification of a clear dural tear, even after Valsalva maneuvers. Therefore, an epidural large fat graft was placed dorsally to cover the entire dural tube, and a subarachnoid spinal drainage positioned for 7 days. Two days after the drainage was removed, the CSF leakage recurred, and an MRI of the lumbosacral spine revealed a circumferential CSF epidural collection spreading through the paraspinal muscles, likely to have been sustained by the vertebral body fragment piercing the dural sac (Figure 5). A second revision surgery was performed. During the procedure the retro-pulsed fragment was removed, after having delicately dissected it from the dural plane. An antero-lateral tear close to the L5 nerve root axilla was then uncovered, by slightly twisting the dural sac. No nerve roots herniation or entrapment were noted. Since primary suturing of the laceration was impossible, a shaped dural patch (Tutopatch^®^), adequately tailored to entirely wrap the dural tube, was positioned on the dural tear, making two cuts to form a slot long enough to accommodate both the L5 nerve roots without retracting them. Then the flaps of the patch folded onto the dorsal dural surface were sutured, without squeezing the nerve roots, but making the patch strictly adherent to the dural tube. No more leakages were noted after repeated intraoperative Valsalva maneuvers, a subarachnoid drainage was positioned, and the post-operative course was uneventful. On the fifth postoperative day the drainage was removed and the day after the patient discharged. The wound was desutured 14 days after the latest procedure, obtaining correct healing. At follow up neither wound dehiscence nor pseudo-meningocele were noted and a complete functional recovery was registered up to 24 months after surgery.

### 4.3. Case Report 3: Coating with Sutured Fat Graft

**Figure 6:**
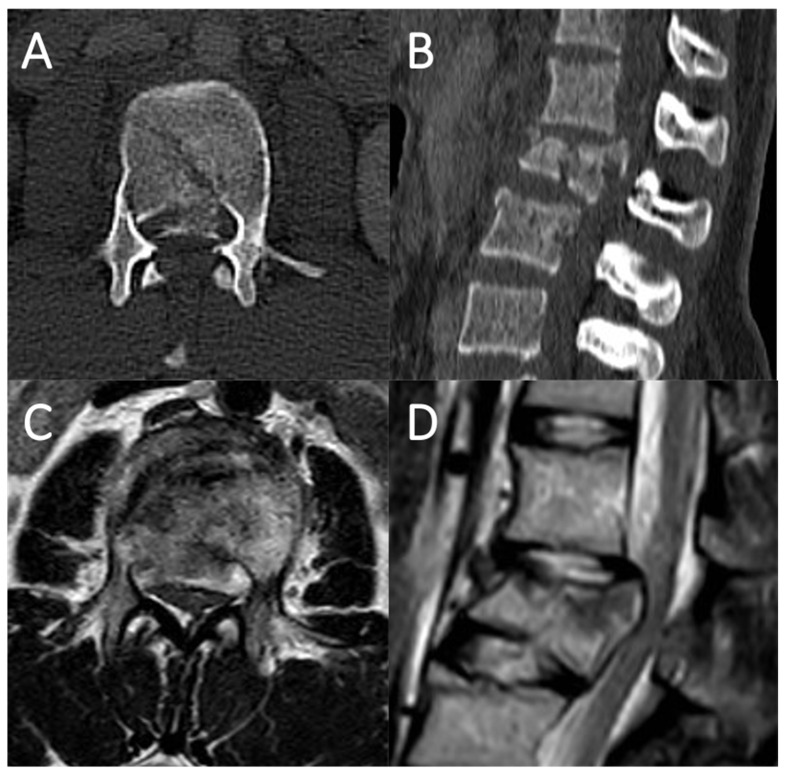
Lumbosacral spine CT-scan ((**A**), axial view; (**B**), sagittal view) and MRI ((**C**), axial view; (**D**), sagittal view) documenting the L2 type A4 fracture with a single vertebral body fragment, which determined a contusion of conus medullaris, encroaching the spinal canal by over 50%.

A 17 year-old female who had fallen from 15 m while attempting suicide was taken to the emergency room. The neurological examination revealed 4/5 muscle strength to the lower limbs with burning dysesthesia in both legs and in the perineal region (ASIA impairment scale: D). Urgent spine CT-scan and MRI documented a L2 type A4 fracture with a single vertebral body fragment which determined a contusion of conus medullaris, encroaching the spinal canal by over 50%. Patient underwent urgent surgery through a T12-L4 screw-rod fixation and L2 laminectomy to provide stability to the fractured vertebra and space to the conus and nerve roots (Figure 6). Intraoperatively, a copious CSF leakage from the ventral aspect of the dural sac was noted, the retro-pulsed vertebral body fragment was impacted, but a clear exposure of the anterior dural laceration was achievable only through a slight twist of the dural sac. A patch (Tutopatch^®^) was adequately tailored, and in its geometric center an autologous fat graft (2.5 cm × 2.5 cm) harvested from the subcutaneous tissue was stitched. After a blunt dissection of the ventral surface of the dural sac from the posterior wall of the vertebral body, the patch was pinched at its angles by suture threads and positioned around the dura, perfectly plugging the dural gap with its stitched fat graft. The entire wrap was then sutured and its watertightness verified with repeated Valsalva maneuvers. A subarachnoid drainage was positioned for 4 days, on the fifth postoperative day it was removed, and the day after the patient was discharged. Wound healing process was uneventful, without any subcutaneous collection up to 18 months after treatment.

## 5. Literature Review

Among the 10 studies yielded by the search strategy describing repair techniques of ventral dural lacerations, a prevalence of primary suturing was noted (*n* = 6/10 papers) [1,3,4,5,6]. In five studies a direct suture was preferred even with posterior approaches by opening the dura on its dorsal surface to perform a trans-dural closure [3,4,5,6,7]. A fat graft plugging the dural opening, sutured to its margins, or stitched to the dura adjacent to the defect and covering all the exposed dura, were other strategies adopted for reaching a watertight repair [8,9,10,11]. With the limitation of two technical notes not referring to specific spinal diseases [8,10], thoracolumbar burst fractures represent the prevalent pathology (*n* = 15/21 cases) determining anterior dural tears [1,4], even considering our three cases. In three patients the ventral dural laceration was a consequence of posterior or anterior decompressions of an ossification of the posterior longitudinal ligament (OPLL) [3,9,11,12], while in the remaining cases such a complication was associated with laminectomy or discectomy for degenerative spine diseases [3,5,8]. In one case, instead, a ventral dural defect was documented as the cause of a spontaneous intracranial hypotension [6]. As noticed in our cases, all the anterior dural lacerations associated with thoracic and lumbar burst fractures were determined by the sharp edges of a vertebral body fractured fragment encroaching the spinal canal >50% [1,4]. Except for our patient with L5 type A3 fracture, all the ventral dural tears associated with vertebral burst fractures reported in the literature were also associated with neurological deficits [1,4]. None of the documented complications was due to the duraplasty, but to the initial pathology which determined the dural laceration itself [1,4,11] (Table 2).

## 6. Discussion

**Figure 7:**
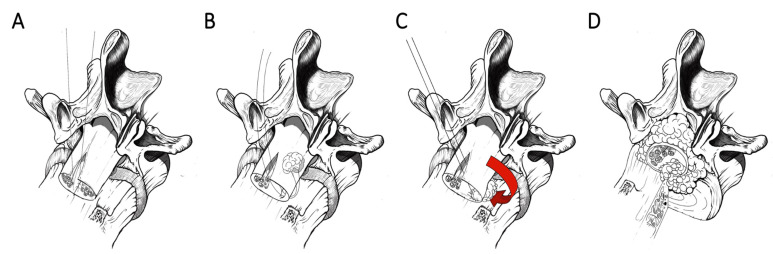
Techniques of anterior dural repair not requiring sacrifice of nerve roots: Trans-dural direct suture (**A**); Piece of fat tied to a suture, inserted into the thecal sac through a second midline durotomy (**B**) and pulled through the dural laceration from the inside out, for plugging antero-lateral dural defects (**C**); Large autologous fat graft, covering all the dural tube and eventually stitched to the intact peripheral dura instead of the dural tear margins (**D**).

Although the clinical significance of dural tears caused by burst fractures remains to be assessed, they can result in diffusion of the blood within the subdural space, CSF leak leading to pseudo-meningocele, meningitis, trapping of herniated nerve roots, and delayed scars involving the neural structures [13]. The literature documents in detail the correlation between posterior element fractures and dural tears and between dural tears and severe neurological deficits [7,14,15,16,17,18,19]. Nevertheless, regarding management and treatment strategies of anterior dural lacerations resulting from fractured vertebral body fragments encroaching the spinal canal, only few reports are available adding little to the topic, especially in terms of techniques for nerve roots preservation and dural sac reconstruction [1,3,4,5,6,7,8,9,11,20]. Although rare, anterior dural tears should be expected in up to 10% of patients with thoracic and lumbar burst fractures, whose fragments encroach the spinal canal enough to pierce the dural sac with their sharp margins [1,21]. Often missed due to incomplete decompression of fracture fragments or epidural bleeding obscuring them [1], such ventral dural tears force surgeons to develop challenging techniques to repair them in a watertight fashion. If a primary suture may be feasible during anterior approaches to the fractured vertebra, their direct closure results demanding from the standard posterior approach commonly used for decompressions and screw-rod fixations. In this latter circumstance most authors performed a midline posterior durotomy with a trans-dural primary suturing of the anterior laceration retracting nerve roots or sacrificing them [3,4,5,6]. Such a technique, particularly risky above the thoracolumbar junction, where the medulla should be retracted or nerve roots cut, appears dangerous, however, below the conus medullaris. In fact, the nerve root manipulation and retraction required by the trans-dural technique could injure such neural structures and trigger subdural bleeding while suturing the defect. Similar considerations should be made even with the double layered repair (epidural and trans-dural) proposed by Lee et al. [9] and with the trans-dural suture of a fat graft adopted by Takai et al. [11]. Without any subdural phase requiring a direct exposure of medulla or nerve roots, the technique described by Black [8] appears as one of the safest and most effective. The described surgical steps, exclusively epidural, together with the use of a large autologous fat graft, covering all the exposed dura and stitched to the intact peripheral dura instead of the dural tear margins, represent indisputable advantages over the other techniques reported in the literature. Inspired by such a concept of restoring a closed dural compartment, maximizing both preservation of neural structures and watertightness, we developed our strategies based on a circumferential epidural coating, creating a single layer enveloping the entire dural tube and not only the anterior dural laceration. Since the dural tear edges are retracted by the bone fragment plunging into the dural sac, often being jagged and macerated, a primary suture may be difficult unless performing an anterior approach for decompression and instrumentation. Therefore, suturing a patch anchored on the peripheral healthy dura appeared as a more cautious strategy [8]. In this sense our coating technique resulted in being even safer, entirely covering the dural sac with a second layer tight enough to stick to it, but not too much to create bottlenecks. Such a thorough refinement of the dural patch is possible thanks to its comfortable closure along the dorsal dural surface, which allows further cutting or tightening of the patch when necessary. Lee et al. performed a double-layer repair, almost entirely wrapping a large dural defect by stitching a Lyoplant patch in the subdural space in addition to another dural patch sutured between the outer dura mater and the vertebral body. The trans-dural exposure of the laceration required T6 and T7 nerve roots to be cut and, despite the choice of a double-layer strategy of sealing, a subarachnoid drainage was positioned for one week [9]. Only in another patient the dural repair was supported by a subarachnoid drainage for 6 days [1,9] and, even if all the other authors did not adopt this, no cases of CSF leak recurrence were reported overall. At the cost of 4 days of subarachnoid drainage to decrease CSF pressure and promote the circumferential sticking of the Tutopatch^®^ on the dural sac [22], our single-layer coating technique revealed itself to be safe and effective, avoiding recurrence of CSF leak and intradural steps which could damage medulla and nerve roots through their manipulation and retraction [4,9]. Whenever the dural laceration is too wide, our epidural coating with a sutured fat graft able to plug the defect may represent an alternative and safer strategy than the double-layer technique (intra- and epidural) suggested by Lee et al., since, similarly to these authors, in our three cases the dural defects were between 18 and 25 mm in length [9]. Antero-lateral tears, instead, pose a technically difficult problem for primary suturing of such inaccessible sites. In addition, antero-lateral dural lacerations that are close to a nerve root are potentially dangerous because the suture may impale the neural fascicles or cause traction or scarring of the nerve root [7,8]. Apart from the case we reported, only Black described and experienced an antero-lateral dural tear close to the nerve root and the relative critical aspects associated with that anatomical location [8]. The insertion of a piece of fat tied to a suture, inserted into the thecal sac through a second midline durotomy and pulled through the dural laceration from the inside out, represents one of the safest strategies for effectively plugging an antero-lateral dural defect. Mainly indicated for sub-centimetric tears close to the origin of the nerve root, where the dura is more fragile, the watertightness of the plug of fat may be reinforced with further peripheral anchoring sutures in addition to the one used to fit it into the dural hole [10] (Figure 7). Even when facing this dural tear location, our technique of coating by straddling the nerve root was a safe and effective alternative to the direct suture documented by Pickett and Blumenkopf [7], to the large fat graft stitched by Black [8] and to the fat plug anchored by Mayfield and Kurokawa [10], preventing CSF from seeping between the Tutopatch^®^ and the dural sac. In our experience, the three types of coating techniques do not require a specific microsurgical experience since the surgical gestures adopted are the same as those used in common maneuvers such as the ligation of medium-caliber vessels.

## 7. Conclusions

Although the recent literature reports encouraging results with suture-less dural repair [23,24], the direct suture remains the preferred method. Therefore, despite the inaccessible location, primary suturing or other strategies ensuring watertightness should be pursued even for sealing off anterior dural tears. Whether single or double-layered, the techniques developed for repairing such lacerations by suturing a dural patch/autologous graft were equally effective in providing a true watertight closure, but different in terms of surgical strain for preserving neural structures. Although with the limitation of few available reports, with clinical and technical descriptions available only in 25 patients including our three cases, epidural single layer repairs proved safer than double layer techniques, which require intradural steps threatening medulla and nerve roots.

The results of this literature review together with our experience in terms of CSF leakage recurrence suggest the importance of immediately aiming at watertightness, with sutured repairs with patch/graft providing optimal results compared to multilayer strategies based on gel-foam and sealant or with patch/graft not sutured, which can be loosened under the effect of CSF pulsation. Nevertheless, future multicentric studies on this topic comparing ventral dural closures with/without sutures and single/multi-layered are needed to verify this hypothesis.

## Figures and Tables

**Table 1:** AOSpine classification system of thoracolumbar spinal fractures.

AOSpine Classification System of Thoracolumbar Spinal Fractures		
Type	Subtype	Description
Type A Compression injuries	A0	Minor, nonstructural fractures Fractures which do not compromise the structural integrity of the spinal column such as transverse process or spinous process fractures
	A1	Wedge-compression Fracture of a single endplate without involvement of the posterior wall of the vertebral body
	A2	Split Fracture of both endplates without involvement of the posterior wall of the vertebral body
	A3	Incomplete burst Fracture with any involvement of the posterior wall; only a single endplate fractured. Vertical fracture of the lamina is usually present and does not constitute a tension band failure
	A4	Complete burst Fracture with any involvement of the posterior wall and both endplates. Vertical fracture of the lamina is usually present and does not constitute a tension band failure
Type B Distraction injuries	B1	Transosseous tension band disruption Monosegmental pure osseous failure of the posterior tension band. Fracture may extend through the pedicle and exit from the posterior aspect of the pars interarticularis or extend through the pedicle through the spinous process before exiting into the soft tissue posteriorly.
	B2	Posterior tension band disruption Bony and/or ligamentary failure of the posterior tension band of the spine together with a type A fracture. Type A fracture should be classified separately
	B3	Hyperextension Injury through the disk or vertebral body leading to a hyperextended position of the spinal column. Commonly seen in ankylotic disorders. Disruption of the anterior longitudinal ligament (ALL) that serves as the anterior tension band of the spine, but there is a posterior hinge preventing further displacement
Type C Displacement or dislocation	C	Displacement or dislocation There are no subtypes because various configurations are possible. Failure of all elements leading to displacement or dislocation in any direction of the fractured segments of the spine.

**Table 2:** Techniques for repairing anterior/antero-lateral dural tears of thoracic and lumbar spine levels.

Authors	N° of Patients	Pathology	Canal Encroachment >50% (y/*n*)	Neurologic Impairment (y/*n*)	Treatment	Location of Dural Tear	Repair Technique	Subarachnoid Drainage (Days)	Complication	Follow-Up (Months)
Mayfield et al. 1975	-	-	-	-	-	Anterior	Fat plug sutured in the defect	-	-	-
Pickett et al. 1989	3	Thoracic and lumbar burst fracture	-	-	Posterior decompression and fusion	Antero-lateral	Direct suture	-	No	-
Carl et al. 2000	6	L2 burst fracture	Y	Y	Anterior fusion	Anterior	Direct suture	-	-	-
		L1 burst fracture	Y	Y	Anterior fusion	Anterior	Direct suture	-	-	-
		L1 burst fracture	Y	Y	Anterior fusion	Anterior	Direct suture	-	-	-
		L2 burst fracture	Y	Y	Anterior fusion	Anterior	Direct suture	-	-	-
		L4 burst fracture	Y	Y	Anterior fusion	Anterior	Direct suture	-	-	-
		T12 burst fracture	Y	Y	Anterior fusion	Anterior	Direct suture	6	CSF leakage in pleural space	24
Black 2002	-	Degenerative spine diseases (thoracic and lumbar)	-	-	Laminectomy and discectomy	Anterior and antero-lateral	Fat pad covering defect and adjacent dura	No	No	-
Huang et al. 2013	5	L2 burst fracture	Y	Y	Posterior decompression and fusion	Anterior	Transdural direct suture	-	No	18
		L1 burst fracture	Y	Y	Posterior decompression and fusion	Anterior	Transdural direct suture	-	No	16
		L2, L3 burst fractures	Y	Y	Posterior decompression and fusion	Anterior	Transdural direct suture	-	Pneumonia, bedsore, wound infection	12
		L1 burst fracture	Y	Y	Posterior decompression and fusion	Anterior	Transdural direct suture	-	Bedsore	9
		L2 burst fracture	Y	Y	Posterior decompression and fusion	Anterior	Transdural direct suture	-	No	8
Choi et al. 2013	4	L4-L5 disc herniation	N	N	Laminectomy and discectomy	Anterior	Transdural direct suture	-	No	6
		L2-L4 bulging and foraminal stenosis	N	N	Laminectomy, discectomy and interspinous device	Anterior	Transdural direct suture	-	No	6
		L4-L5 disc herniation	N	N	Laminectomy, discectomy and interspinous device	Anterior	Transdural direct suture	-	No	6
		L1-L2 disc herniation and OPLL	N	N	Anterior decompression and fusion	Anterior	Transdural direct suture	-	No	6
Schievink et al. 2016	1	T9-T10 ventral dural defect in spontaneous intracranial hypotension	N	N	-	Anterior	Transdural direct suture	No	No	12
Lee et al. 2016	1	Thoracic OPLL	-	Y	Posterior decompression and fusion	Anterior	Double layered (epidural and transdural)	7	No	16
Nakhla et al. 2017	1	Grade II spondylolisthesis L4-L5	N	N	Posterior lumbar interbody fusion	Anterior	Transdural direct suture	No	No	6
Takai et al. 2018	1	T12-L1 osteophyte	N	N	Laminoplasty	Anterior	Transdural suture of fat graft	-	Subdural hematoma	24
